# Phosphorylation of Lamin A/C regulates the structural integrity of the nuclear envelope

**DOI:** 10.1016/j.jbc.2024.108033

**Published:** 2024-11-28

**Authors:** Shuaiyu Liu, Fangyuan Xiong, Zhen Dou, Lingluo Chu, Yihan Yao, Ming Wang, Xuebiao Yao, Xing Liu, Zhikai Wang

**Affiliations:** 1 MOE Key Laboratory for Cellular Dynamics, Center for Advanced Interdisciplinary Science and Biomedicine of IHM, Hefei National Research Center for Interdisciplinary Sciences at the Microscale, University of Science and Technology of China, Hefei, China; 2 Anhui Key Laboratory of Cellular Dynamics and Chemical Biology, University of Science and Technology of China, Hefei, China; 3 Hong Kong University of Science and Technology (Guangzhou), Guangzhou, China; 4 Cambridge University Department of Chemistry, Cambridge, UK

**Keywords:** mitosis, Lamin A, phosphorylation, CDK1, nuclear integrity

## Abstract

Dynamic disassembly and reconstruction of the nuclear lamina during entry and exit of mitosis, respectively, are pivotal steps in the proliferation of higher eukaryotic cells. Although numerous post-translational modifications of lamin proteins have been identified, key factors driving the nuclear lamina dynamics remain elusive. Here we identified CDK1-elicited phosphorylation sites on endogenous Lamin A/C and characterized their functions in regulation of the nuclear lamina. Specifically, mass spectrometry revealed CDK1-mediated phosphorylation of Lamin A/C at the N-terminal Thr19/Ser22 and the C-terminal Ser390/Ser392 during mitosis. Importantly, the phospho-mimicking 4D mutant T19D/S22D/S390D/S392D completely disrupted Lamin A filamentous structure in interphase cells. Conversely, the non-phosphorylatable mutant T19A/S22A and especially the 4A mutant T19A/S22A/S390A/S392A protected Lamin A from depolymerization during mitosis. These results suggest that phosphorylation and dephosphorylation of both N- and C-terminal sites regulate the nuclear lamina dynamics. Engineering the non-phosphorylatable mutant T19A/S22A into the endogenous *LMNA* gene resulted in nuclear abnormalities and micronucleus formation during telophase. Perturbation of the Lamin A phosphorylation is shown to prevent proper nuclear envelope dynamics and impair nuclear integrity. These findings reveal a previously undefined link between the CDK1-elicited Lamin A phosphorylation dynamics, nuclear envelope plasticity, and genomic stability during the cell cycle.

The nuclear lamina is located beneath the inner nuclear membrane and serves as the structural scaffold for the nuclear envelope (1). Lamin proteins are the main structural components of the nuclear lamina, forming a dense filamentous meshwork that varies in thickness from 10 nm to 30 nm in different cell types (2). In mammalian cells, lamins are classified as type V intermediate filament proteins and are subdivided into A-type and B-type subgroups. The A-type lamins include Lamin A and Lamin C which are encoded by the same *LMNA* gene, whereas the two major B-type lamins, Lamin B1 and Lamin B2, are encoded by the *LMNB1* and *LMNB2* genes, respectively (3). Common to most intermediate filament proteins, these lamins consist of three structural regions: an N-terminal head domain, a coiled-coil central rod domain, and a C-terminal tail domain. The C-terminal tail domain contains a nuclear localization signal, an immunoglobulin-like (Ig) fold, and, except for Lamin C, a distal CaaX motif that undergoes farnesylation and methyl esterification (4). Functionally, lamins play roles in defining nuclear shape, mechanosignaling, stress responses, nuclear organization, chromatin stabilization, gene expression regulation, DNA replication and repair, and cell cycle progression (5).

Several studies have reported that *LMNA* mutations and the resulting alterations in Lamin A/C proteins or their modifications are linked to the progression of several diseases (6). These so-called laminopathies include muscular dystrophy, cardiomyopathy, and Hutchinson-Gilford progeria (HGPS), a premature aging disorder (7). HGPS, for example, is a rare and fatal childhood disease associated with heterozygous mutations in the *LMNA* gene. These mutations create a cryptic splice site resulting in a deletion of 50 amino acids near the C-terminus of Lamin A (8, 9). This deletion removes the Zmpste24 cleavage site and generates a permanently farnesylated, but functionally dominant, mutant of pre-Lamin A known as progerin. The median age of the affected patients at death is 13.4 years, usually due to coronary artery disease (10).

Protein phosphorylation serves as a central regulatory mechanism that modulates Lamin A/C function, either during mitosis or under specific physiological/pathological conditions. The kinases involved include CDK1 (11, 12, 13), CDK5 (14), protein kinase C (PKC) (15), AKT1/2 (16, 17, 18, 19, 20), and ATR (21, 22, 23). A recent study demonstrated that phosphorylation of nucleoplasmic Lamin A/C at Ser22 (pSer22) promotes its binding to a subset of active enhancers to facilitate gene transcription (24). Lamin A/C phosphorylation has also been reported to respond to extracellular mechanical stress, which plays a role in maintaining nuclear stability (25, 26). In addition, phosphorylation and dephosphorylation of lamins control mitotic disassembly and post-mitotic reconstruction of the nuclear envelope, respectively. Phosphorylation of Lamin A at Ser22 and Ser392 by CDK1 leads to its disassembly as the cell enters mitosis (27). Localization of the Repo-Man/PP1 complex to the mitotically segregated chromatin is essential for Lamin A/C-pSer22 dephosphorylation which initiates the nuclear envelope reassembly (28, 29, 30). Previous studies have predominantly used *in vitro* phosphorylation assays and other biochemical experiments to demonstrate the post-translational modification and the biochemical functions of Lamin A/C. However, the precise regulations and functions of these Lamin A/C phosphorylation sites in living cells remain to be evaluated.

In this study, we used immunoprecipitation/mass spectrometry, CRISPR-mediated gene editing, live cell imaging, and immunofluorescence staining with the phosphorylation site-specific antibodies to assess the phospho-regulation of Lamin A/C during mitosis. We discovered that Thr19/Ser22 at the N-terminus and Ser390/Ser392 at the C-terminus are phosphorylated during mitosis. Interphase cells expressing the phospho-mimicking mutant, either T19D/S22D or S390D/S392D, showed a distribution of a large amount of the protein in the nucleoplasm. In addition, the mutation of all four amino acids to non-phosphorylatable alanine, but not the N- or C-terminal duos, prevented the lamin dissolution during mitosis. Our results suggest that phosphorylation of either the N- or C-terminal duo allows the mitotic disassembly of the nuclear lamina, whereas dephosphorylation of all four residues is required for proper nuclear envelope reconstruction. Nevertheless, interphase cells expressing the N-terminal T19A/S22A mutant exhibited significant abnormalities in nuclear morphology and an increased incidence of micronucleus. This indicates that dynamic phosphorylation during mitosis and dephosphorylation post mitosis of Thr19 and Ser22 are indispensable for the maintenance of normal nucleus function.

## Results

### Phosphorylation of Lamin A at the N- and C-termini relocates to the nucleoplasm

**Figure 1:**
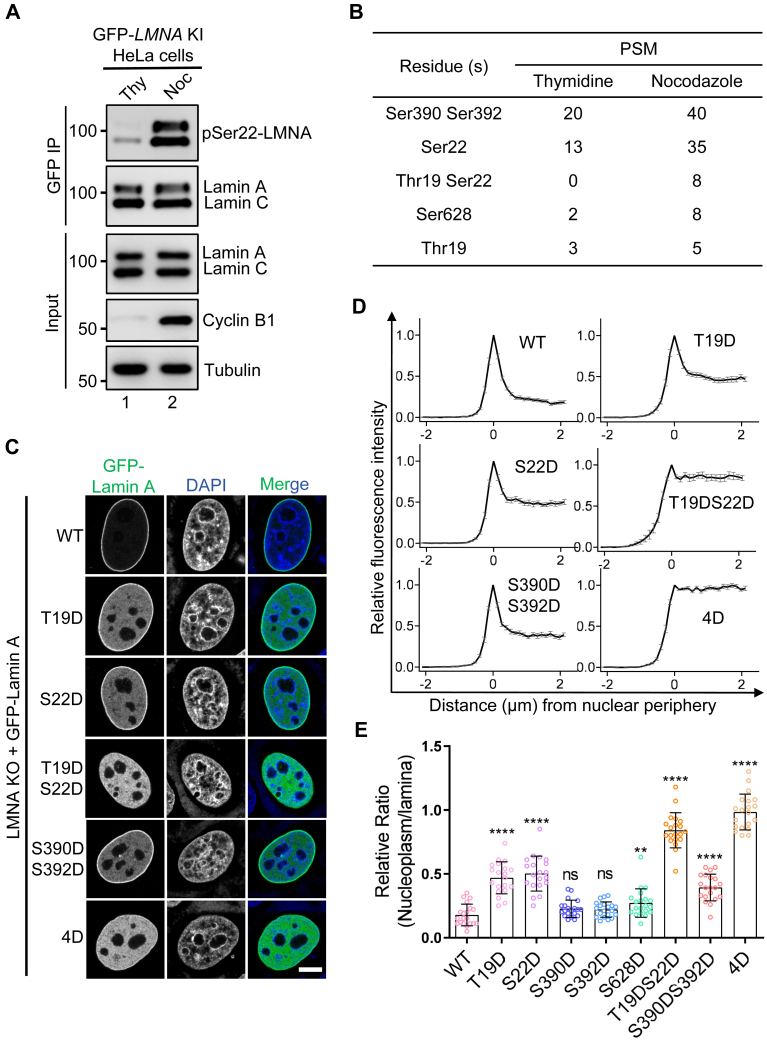
**Phosphorylation of Lamin-A N- and C-terminus perturbs the correct subcellular localization**. *A*, endogenously engineered HeLa cells expressing GFP-Lamin A were synchronized at G1/S phase and mitosis, using thymidine and nocodazole, respectively. The cell lysates and anti-GFP immunoprecipitates were applied to SDS-PAGE and Western blotting with pSer22-LMNA, anti-Lamin A/C, anti-Cyclin B1, and anti-Tubulin antibodies. *B*, the anti-GFP immunoprecipitates in (*A*) were applied to mass spectrometric analysis (LC-MS/MS) for phosphorylation sites. Numbers of peptide-to-spectrum matches (PSM) for the identified phosphorylation sites were listed. *C*, representative HeLa cells expressing GFP-tagged Lamin A-WT and the mutants, in which endogenous *LMNA* was knocked out (KO), were fluorescently imaged and shown. Scale bar, 10 μm. *D*, line scan analysis was performed for GFP-Lamin A-WT and the mutants in (*C*). The immunofluorescence intensity along the 4-μm lines that were drawn across the nuclear periphery (0 μm) was statistically analyzed. The negative x coordinates indicate positions outside the nucleus. n = 20. *E*, ratio of the immunofluorescence signal in the nucleoplasm at 2 μm and the nuclear lamina was statistically analyzed for GFP-Lamin A in (*C* and *D*). Data are presented as mean ± S.D. One-way ANOVA with Dunnett’s multiple comparison test. ns, not significant; ∗∗*p* < 0.01; ∗∗∗∗*p* < 0.0001.

To systemically identify the specific phosphorylation sites of Lamin A/C during mitosis at the endogenous protein level, we used clustered regularly interspaced short palindromic repeats (CRISPR)-Cas9 to engineer endogenous *LMNA* fused with a monomeric enhanced GFP at the N-terminus in HeLa cells. The fusion protein in a selected subclone was tested to express at a level comparable to that of endogenous Lamin A/C (Fig. S1, *A*–*C*). Real-time imaging analyses show that the engineered GFP-Lamin A/C normally undergoes disassembly and reassembly at entry and exit of mitosis, respectively (Fig. S1*D*). Subsequently, mass spectrometric analyses were conducted for the GFP-Lamin A/C immunoprecipitates purified from synchronized HeLa cells at interphase and mitosis, respectively, which identified characteristic phosphorylation of Thr19, Ser22, Ser390, Ser392, and Ser628 (unique to Lamin A) in the mitotic sample (Figs. 1, *A* and *B* and S2, *A*–*C*).

To evaluate the functions of these phosphorylated sites in Lamin A/C assembly, we generated a HeLa cell line with the endogenous *LMNA* inactivated by CRISPR-Cas9 (Fig. S1, *E* and *F*) and transfected the cells to express the phosphorylation-mimicking Lamin A mutants. Compared to the wild-type (WT) GFP-Lamin A, the T19D and S22D single mutants exhibited a notable distribution in the nucleoplasm, while the T19DS22D double mutant exhibited an even greater nucleoplasmic dispersion (Fig. 1, *C* and *D*). In contrast, the S390D, S392D, and S628D single mutations had minimal effects (Fig. S2, *D* and *E*), while the S390DS392D double mutation resulted in an increase in the nucleoplasmic fluorescence (Fig. 1, *C* and *D*). Moreover, the 4D mutant (T19D/S22D/S390D/S392D) exhibited a complete diffusion within the nucleoplasm (Fig. 1*E*). Thus, we conclude that both N- and C-terminal phosphorylation sites are involved in the depolymerization of Lamin A during mitosis, with the N-terminal phosphorylation sites playing a crucial role in this process.

### Thr19 of Lamin A/C is phosphorylated in mitosis

**Figure 2:**
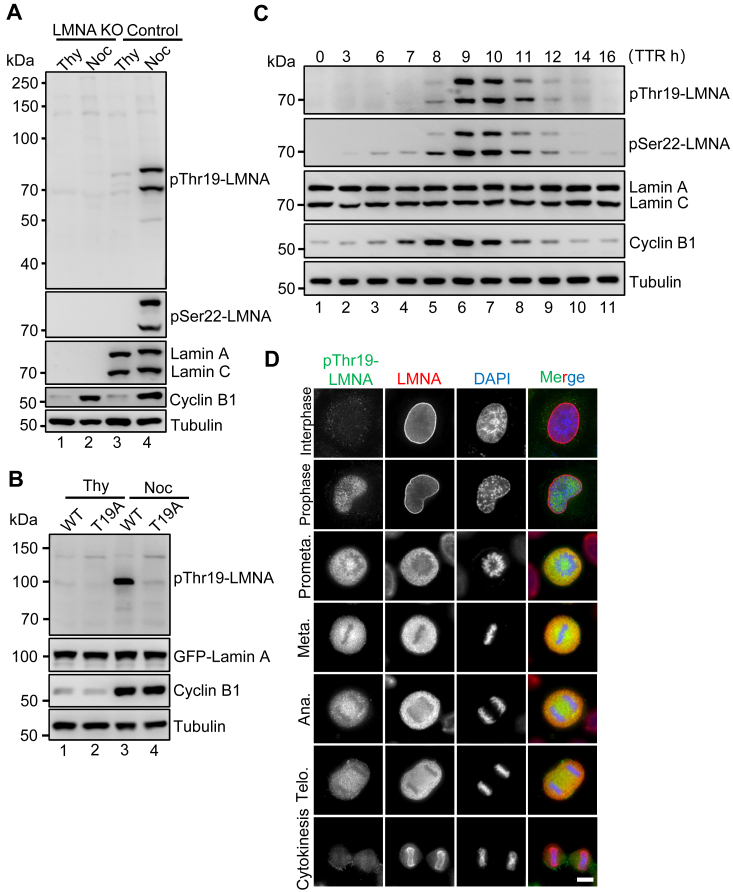
**Thr19 of Lamin A/C is phosphorylated in mitosis**. *A*, unperturbed (control) and *LMNA* KO HeLa cells that were synchronized at G1/S phase and mitosis, using thymidine and nocodazole, respectively, were lysed and subjected to SDS-PAGE and Western blotting analysis using the indicated antibodies. *B*, HeLa cells stably expressing GFP-Lamin A-WT and the T19A mutant, in which endogenous *LMNA* was inactivated, were synchronized at the G1/S phase and mitosis with thymidine and nocodazole, respectively. Subsequently, the cells were collected and lysed for Western blotting analysis using the indicated antibodies. *C*, temporal profiling of Lamin A/C Thr19 and Ser22 phosphorylation throughout the cell cycle. HeLa cells that were synchronized at G1/S phase by double thymidine block, were washed to release the block for the indicated time. The cell lysate samples were resolved by SDS-PAGE and subsequently analyzed by Western blotting using the indicated antibodies. TTR, double thymidine release. *D*, HeLa cells were immunofluorescently stained for pThr19-Lamin A/C (*green*) and Lamin A/C (*red*), and nucleus (DAPI, *blue*). Representative images of the cells at the various cell cycle stages were shown. It is noteworthy that the pThr19 signal is robust in metaphase cells but markedly diminished in telophase cells. Scale bar, 10 μm.

In light of the pivotal function of the N-terminal phosphorylation sites in Lamin A disassembly, and the extensive research on Ser22 phosphorylation (12, 13), we seek to gain a deeper understanding of the temporal and spatial dynamics of Thr19 phosphorylation. To probe Thr19 phosphorylation dynamics, whole cell lysates of control and the *LMNA* knockout HeLa cells that were synchronized in G1/S interphase and mitosis were subjected to SDS-PAGE electrophoresis and immunoblotting with a home-made Lamin A/C-Thr19 phosphorylation-specific antibody (pThr19-LMNA). A robust and specific signal showed up exclusively in the control mitotic cells, which is coincident with Lamin A/C-Ser22 phosphorylation (Fig. 2*A*). Reliability of the pThr19-LMNA antibody was further demonstrated by Western blotting for the Lamin A-T19A mutant (Fig. 2*B*), where the cell lysates were extracted from HeLa cell lines stably expressing GFP-tagged WT Lamin A and the T19A mutant, with endogenous Lamin A/C depleted by CRISPR-Cas9 (Fig. S3*A*). These findings show that Thr19 of Lamin A/C is phosphorylated during mitosis.

To evaluate the temporal dynamics of Thr19 phosphorylation across the cell cycle, we synchronized HeLa cells at G1/S boundary by double thymidine block and released the arrest to the indicated time points, for immunoblotting with the pThr19-LMNA and pSer22-LMNA antibodies. The emergence of Cyclin B1 marked the transition to the G2 phase, which occurred ∼8 h after the release and persisted until the exit of mitosis (∼11 h). The temporal distribution of pThr19-LMNA and pSer22-LMNA signals mirrored that of Cyclin B1, reaching a peak at approximately 9 h post-release (Fig. 2*C*). This finding implied that the phosphorylation of Thr19 and Ser22 might be generated by CDK1-Cyclin B1.

Having demonstrated the specificity of the pThr19-LMNA antibody and the temporal presence of the modification, we proceeded to investigate the subcellular localization of the phosphorylated Lamin A/C in unperturbed HeLa cells. As shown in Figure 2*D*, pThr19-LMNA signal was minimal in interphase cells but became apparent in the nucleoplasm of prophase when chromatin DNA started to condense. This signal lasted until the cells exited mitosis, a time window coinciding with Cyclin B1 expression and CDK1 activation. The temporal distribution of Ser22 phosphorylation exhibited a similar pattern to that of pThr19, although pSer22 maintained a certain level of abundance in the nucleoplasm of interphase cells (Fig. S3*B*). This finding is consistent with previous research that nucleoplasmic pSer22-LMNA associates with active enhancers in interphase cells to regulate gene activation (24). The fluorescence intensities of both pThr19 and pSer22 reached their highest levels at metaphase, which correlated with the complete depolymerization of Lamin A/C throughout the cell cycle (Fig. S3*C*). These observations suggest that phosphorylation at Thr19 and Ser22 occurs in mitosis to promote Lamin A depolymerization.

### Thr19 of Lamin A/C is a substrate of CDK1 kinase

**Figure 3:**
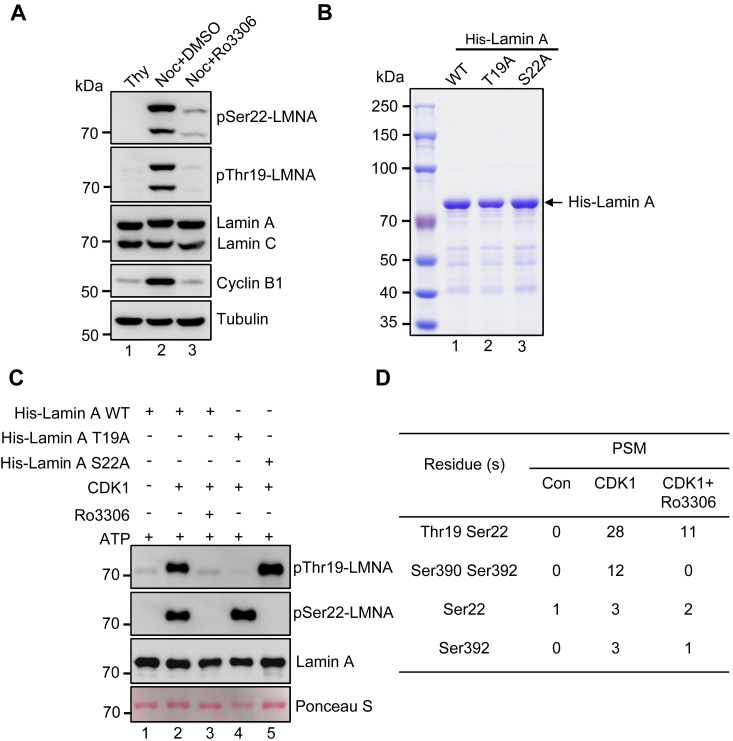
**Thr19 of Lamin A/C is a substrate of CDK1 kinase.***A*, nocodazole-arrested miotic HeLa cells were additionally treated with DMSO or Ro3306 for 30 min. Subsequently, the cells were harvested and the lysates were subjected to Western blotting analysis using the indicated antibodies, in which thymidine-arrested G1/S phase cells were included as control. *B*, quality assessment of recombinant 6xHis-tagged Lamin A-WT, T19A, and S22A mutants, which were purified from bacteria for *in vitro* kinase assay, by means of SDS-PAGE electrophoresis and Coomassie Brilliant Blue staining. *C*, *in vitro* phosphorylation of the recombinant proteins as in (*B*), by CDK1 kinase in the absence or presence of the kinase inhibitor Ro3306, was conducted and applied to Western blotting analyses with the indicated antibodies. Ponceau S staining of the Lamin A proteins was shown as loading control. *D*, mass spectrometry (LC-MS/MS) was employed to analyze the phosphorylated residues of the CDK1-treated Lamin A.

To ascertain whether Thr19 is a *bona fide* substrate of CDK1, we treated nocodazole-arrested mitotic cells with DMSO or Ro3306, a CDK1 kinase-specific inhibitor. Compared to the DMSO-treated cells, Ro3306 treatment resulted in substantial suppression of pThr19-LMNA and pSer22-LMNA signals (Fig. 3*A*). In addition, an *in vitro* phosphorylation assay was conducted with bacterially recombinant His-tagged Lamin A-WT, as well as the T19A and S22A mutant proteins (Fig. 3*B*). As shown in Figure 3*C*, T19A and S22A mutations completely suppressed the CDK1-mediated phosphorylation of Thr19 and Ser22, respectively. Addition of Ro3306 to the reaction of His-Lamin A-WT resulted in an elimination of phosphorylation at both sites (Fig. 3*C*). Mass spectrometric analyses of the *in vitro* phosphorylated samples further validated that Thr19, Ser22, Ser390, and Ser392 were phosphorylated by CDK1, and that CDK1 chemical inhibitor Ro3306 treatment greatly reduced these phosphorylations (Fig. 3*D*). These findings corroborate with the mass spectrometric data in cells and confirm that Thr19 of Lamin A is a *bona fide* substrate of CDK1 during mitosis.

### T19A and S22A mutants delay Lamin A depolymerization

**Figure 4:**
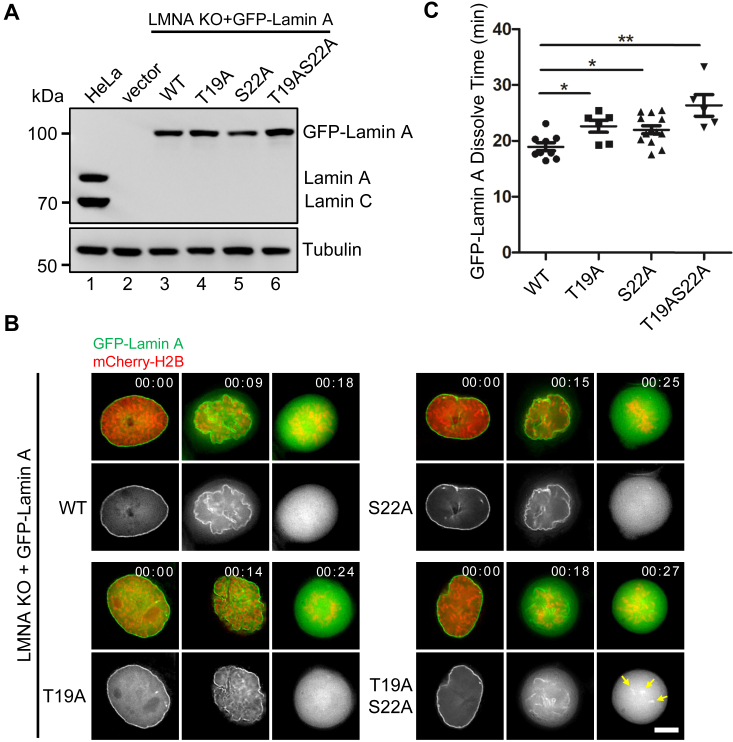
**The non-phosphorylatable mutants of T19A and S22A lead to depolymerization delay of Lamin A**. *A*, lentiviruses expressing GFP-Lamin A WT and the mutants were used to infect the *LMNA* KO HeLa cells. Western blotting analysis was employed to assess the expression levels of the endogenous Lamin A proteins. *B*, real-time imaging of the *LMNA* KO HeLa cells stably expressing GFP-tagged Lamin A-WT or the mutants, in which chromosomes were visualized by the co-expressing mCherry-H2B. Scale bar, 10 μm. *C*, quantitative analysis of the Lamin A dissolution time during mitotic entry in (*B*). A total of approximately 25 cells were analyzed for each group. Data are presented as mean ± S.D. One-way ANOVA with Dunnett’s multiple comparison test. ∗*p* > 0.05; ∗∗*p* < 0.01.

Prior biochemical and immunofluorescence experiments have demonstrated that phosphorylation of Ser22 and Ser392 facilitates Lamin A depolymerization during mitosis (13). Our results confirmed the phosphorylation of Thr19 and Ser22 at the N-terminus and that of Ser390 and Ser292 at the C-terminus of endogenous Lamin A/C. To ascertain whether and how phosphorylation of Thr19 and Ser22 regulates Lamin A disassembly in mitosis, we transiently expressed GFP-tagged Lamin A-WT, T19A, S22A, and T19AS22A in HeLa cells with endogenous Lamin A/C depleted. The expression levels of these constructs were validated to be comparable to that of endogenous Lamin A/C (Fig. 4*A*). Real-time imaging demonstrated that GFP-Lamin A-WT readily underwent depolymerization at the entry of mitosis and became fully dispersed by prometaphase (Fig. 4*B*). However, both the T19A and S22A single mutants exhibited delays in Lamin A depolymerization, and the T19AS22A double mutant further prolonged the time required for a full dissolution (Fig. 4, *B* and *C*). These findings suggest that phosphorylation of Thr19 and Ser22 plays a pivotal role in enabling the expeditious and effective depolymerization of Lamin A during mitosis.

### Expression of engineered *LMNA* T19A and S22A mutants causes nuclear lamina defects

**Figure 5:**
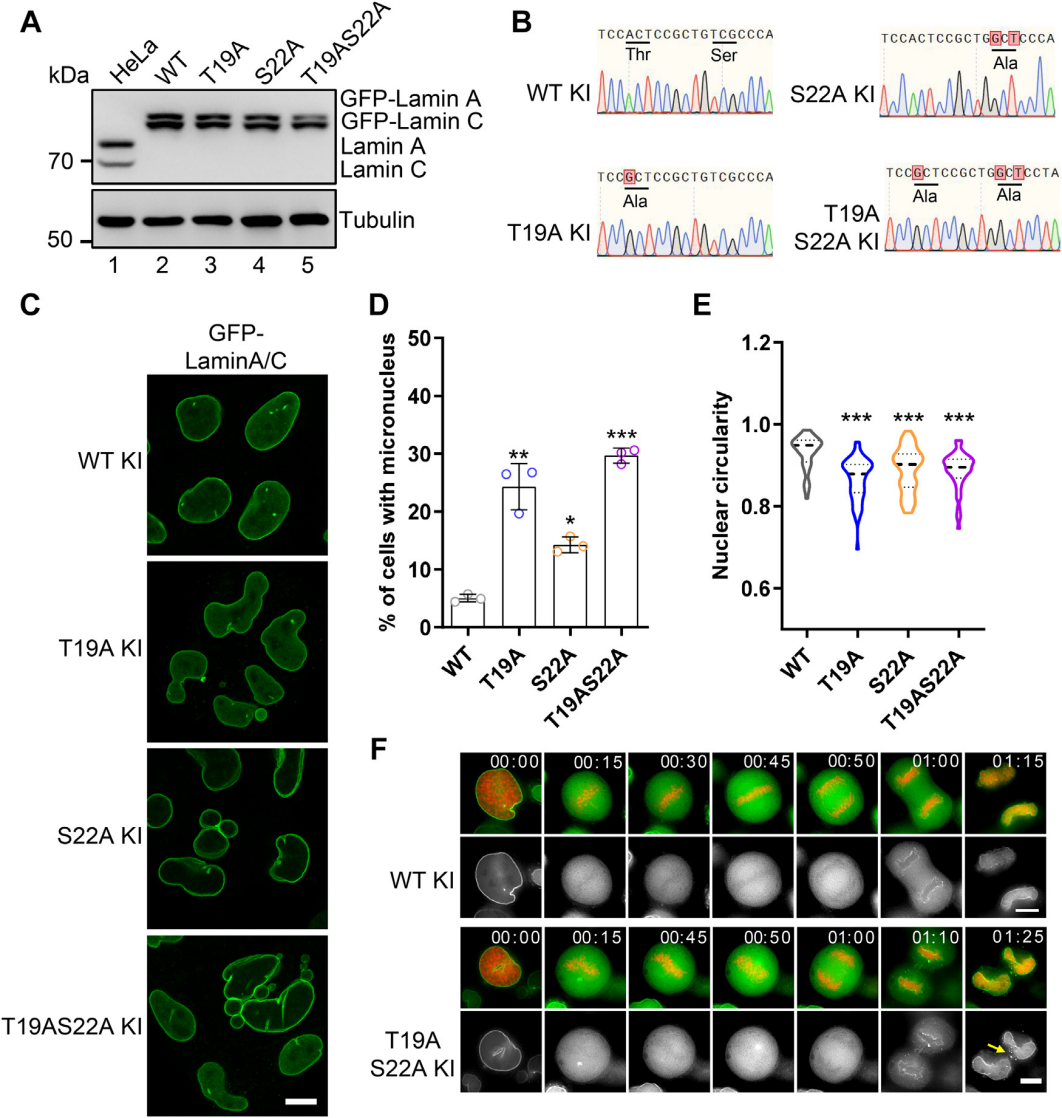
**The non-phosphorylatable mutants of T19A and S22A lead to nuclear lamina defects**. *A*, HeLa cells, in which endogenous *LMNA* was mutated and GFP-tagged by CRISPR, were lysed and applied to Western blotting using an anti-Lamin A/C antibody. *B*, genotyping sequences of Lamin A-WT and the mutants for the knock-in (KI) single-cell clones as in (*A*) were shown. *C*, representative immunofluorescence images of GFP-Lamin A/C-WT and the mutants showing the nuclear morphology of the single-cell clones as in (*A* and *B*) are presented. Scale bar, 10 μm. *D*, quantification of the GFP-Lamin A/C WT and the mutants expressing cells with one or more micronucleus is shown. Data are presented as mean ± S.D. One-way ANOVA with Dunnett’s multiple comparison test. ∗*p* > 0.05; ∗∗*p* < 0.01; ∗∗∗*p* < 0.001. *E*, violin plot showing the statistics of nuclear circularity for the cells as in (*C*) is presented. A minimum of 200 cells were analyzed. One-way ANOVA with Dunnett’s multiple comparison test. ∗∗∗*p* < 0.001. *F*, representative time-lapse images of HeLa cells expressing GFP-Lamin A-WT and the T19AS22A mutant (*green*) are showing. The chromosomes were visualized by the co-expressing mCherry-H2B (*red*). The arrowhead indicates the presence of anomalous polymerized Lamin A filaments in the vicinity of the chromosomes.

We subsequently investigated the phenotypic consequences of the non-phosphorylatable mutants at the endogenous protein expression level over multiple cell cycles. To this end, we employed CRISPR-Cas9 to mutate the *LMNA* gene, thereby generating HeLa knock-in cell lines that express GFP-tagged Lamin A/C-T19A, S22A, and T19AS22A mutants. Western blot analysis demonstrated that the expression levels of these mutants were comparable to that of the Lamin A/C-WT (Fig. 5*A*). Furthermore, Sanger sequencing demonstrated that all mutant cell lines were homozygous (Fig. 5*B*).

Immunofluorescence imaging analysis of the mutants expressing cells revealed a prevalence of nuclear abnormalities, including lamina invagination, micronucleus, and blebbing (Fig. 5*C*). The quantification analysis indicated a notable increase in the proportion of mutant cells displaying micronucleus in comparison to those expressing Lamin A-WT (Fig. 5*D*). Moreover, a reduction in nuclear "roundness" (4π ⨯ Area/Perimeter^2^) was observed for the mutants expressing cells, indicating an increased prevalence of irregularly shaped nucleus (Fig. 5*E*). To ascertain the underlying causes of these aberrant nuclear morphologies, we conducted time-lapse imaging of the entire mitosis process in cells expressing GFP-Lamin A-WT and the mutants. Compared to Lamin A-WT which underwent re-polymerization around chromosomes during telophase, Lamin A-T19AS22A presented several abnormal reassembly filaments that detached from the chromosomes (Fig. 5*F*, yellow arrow). This finding is consistent with previous studies that suppression of lamin dephosphorylation resulted in abnormalities in nuclear morphology at mitotic exit (28). Thus, these results underscore the pivotal role of the phosphorylation/dephosphorylation-regulated polymerization/depolymerization of the nuclear lamina in safeguarding genome integrity and sustaining optimal nuclear morphology.

### The N- and C-terminal phosphorylations synergistically promote Lamin A depolymerization

**Figure 6:**
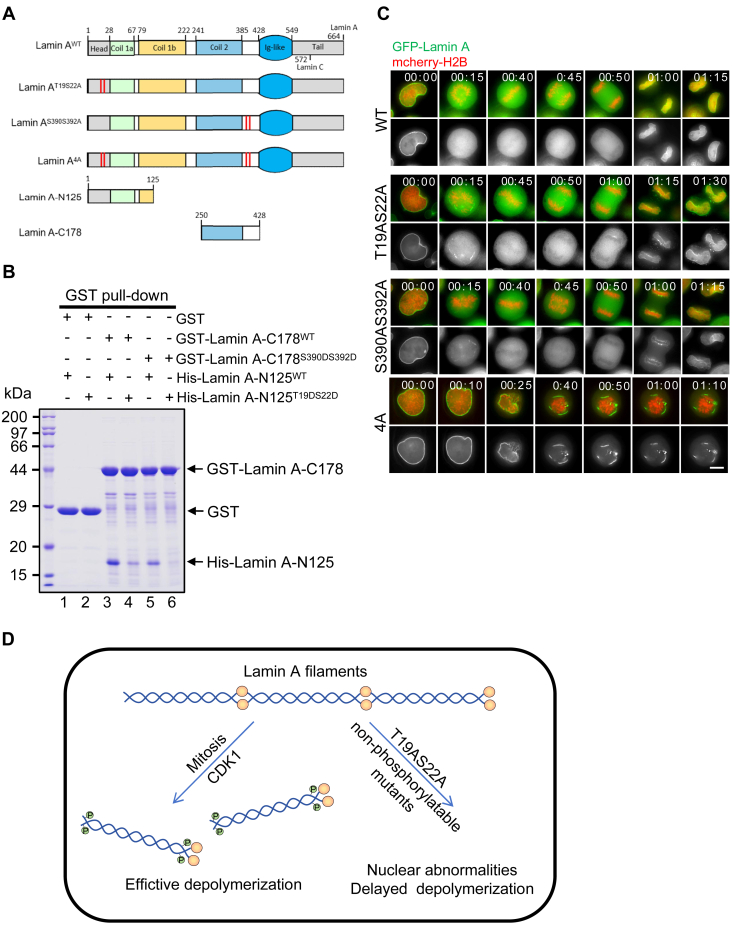
**The N-terminal and C-terminal phosphorylations promote the depolymerization of Lamin A**. *A*, schematic showing the domain organization and the mutation sites of Lamin A-WT and the mutant proteins is presented. *B*, recombinant GST and GST-tagged Lamin-A-C178-WT and the mutant, that were attached to the glutathione microbeads, were used as matrices to absorb Lamin A-N125-WT and the mutant. Following three washes, the eluates from the microbeads were applied to SDS-PAGE electrophoresis and Coomassie Brilliant Blue staining. *C*, real-time imaging of the *LMNA* KO HeLa cells stably expressing GFP-tagged Lamin A-WT and the mutants is representatively showing. The chromosomes were visualized by the co-expressing mCherry-H2B. Scale bar, 10 μm. *D*, working model accounting for the function of CDK1 phosphorylation of Lamin A is shown.

The mass spectrometric analysis of endogenous Lamin A/C revealed an elevation of Ser390 and Ser392 phosphorylations in mitosis, residues situated in the C-terminal tail of Lamin A/C (Fig. 1*B*). The basic unit of lamins is the protein dimer, which forms protofilaments in a head-to-tail manner for the ultimate higher-order assemblies (2). To determine whether phosphorylation at the N- and C-terminal regions affects the assembly of lamin A filaments, an *in vitro* binding assay was performed using recombinant Lamin A-N125 (1–125 aa) and Lamin A-C178 (250–428 aa) (Fig. 6*A*). To that end, GST and GST-tagged Lamin A-C178 were attached to microbeads as matrices to absorb His-tagged Lamin A-N125, and a strong interaction between the two Lamin-A fragments was revealed (Fig. S4*A*). This binding is negatively regulated, to some extent, by salt concentration (Fig. S4*B*), suggesting that electrostatic interactions are involved in the assembly of Lamin A filaments. Subsequently, the phosphorylation-mimicking mutants of the Lamin-A fragments were purified for the same pull-down assay (Fig. S4*C*). It is noteworthy that T19DS22D (lane 4) mutations of Lamin A-N125 and S390DS392D (lane 5) mutations of Lamin A-C178 greatly weakened the interaction, with the T19DS22D and S390DS392D (lane 6) mutants showing no interaction at all (Fig. 6*B*). This finding is consistent with the localization results observed in the cell (Fig. 1*C*), implying that phosphorylation of both N- and C-terminal duos promotes full disassembly of Lamin A/C. To elucidate the function of these phosphorylation sites in Lamin A disassembly within live miotic cells, we constructed GFP-tagged Lamin A-WT, T19AS22A, S390AS392A, and T19AS22AS390AS392A (4A) for time-lapse imaging (Fig. 6*A*). The disassembly kinetics of the C-terminal mutant was comparable to that of Lamin A-WT. Mutation of the N-terminal phosphorylation sites resulted in a slight retardation of disassembly (Fig. 6*C*). However, the concurrent mutation of both N- and C-terminal phosphorylation sites led to a severe deficiency in disassembly, ultimately arresting the cell in mitosis (Fig. 6*C*). These findings suggest that phosphorylation at both the N- and C-terminal residues is essential for effective depolymerization of Lamin A during mitotic progression.

## Discussion

In 1990, immunofluorescence studies were conducted on the human GFP-tagged Lamin A mutants that were transfected into CHO cells, which revealed that the S22A and S392A mutants can impede its depolymerization during mitosis (13). In recent years, numerous studies have employed mass spectrometry and biochemical experiments to demonstrate that multiple phosphorylation sites promote Lamin A depolymerization (11, 15, 31, 32, 33). Nevertheless, the precise function of these sites within live mitotic cells at the endogenous protein level remains unclear. In this study, we demonstrated that during mitosis, the efficient depolymerization of Lamin A is governed by CDK1-mediated phosphorylation of both its N- and C-terminal regions. Engineering the non-phosphorylatable mutant T19A/S22A into the endogenous *LMNA* gene resulted in nuclear abnormalities and delayed depolymerization (Fig. 6*D*).

Post-translational modifications are of critical importance in the regulation of Lamin A function (5, 6, 34, 35). Recent research indicates that disruption of MOF-mediated acetylation of Lamin A enhances its solubility, disrupts the dynamics of phosphorylation, and weakens the mechanostability of the nucleus. Furthermore, acetylation functions as a mechanism to maintain nuclear structure and genome integrity (36). Hyper-SUMOylation of Lamin A has been shown to result in nuclear structure alterations that are similar to those observed in laminopathies (37). The present study demonstrates that cells harboring endogenous point mutations in N-terminal phosphorylation sites exhibit significant abnormalities in nuclear morphology, being accompanied by an increased occurrence of micronucleus. This finding is corroborated by another recent study (38). The integrity of the nuclear membrane is vital for genome stability (39, 40, 41). Recent research has demonstrated that DNA damage can induce nuclear envelope rupture by promoting nuclear lamina breakdown *via* the DNA damage response kinase ATR, with lamins emerging as a novel target of ATR signaling (21, 22, 23). Moreover, the nucleoplasmic pool of Lamin C rapidly accumulates at rupture sites to facilitate repair (42, 43). Thus, these findings highlight the significance of Lamin A phosphorylation in preserving nuclear morphology and genome stability.

Mutations in the *LMNA* gene that result in alterations in phosphorylation are associated with the pathogenesis of several diseases. For example, a defect in phosphorylation of Lamin A at Ser458 due to *LMNA* gene mutations has been associated with myopathy (19). Moreover, alterations in the N-terminal phosphorylation of Lamin A/C, in conjunction with a muscle-specific phosphorylation partner, may contribute to the pathogenesis of Emery-Dreifuss muscular dystrophy and limb-girdle muscular dystrophy 1B (44). In addition, the pathogenic mutation of Ser22 to Leu has been associated with the development of dilated cardiomyopathy (45). The abnormalities in nuclear morphology and genomic instability observed in the cells expressing Lamin A N-terminal phosphorylation mutant in this study may serve as a potential mechanism for the related diseases. Further research is required to gain a deeper understanding of the mechanisms underlying the laminopathies to facilitate the development of more effective therapeutic strategies.

## Experimental procedures

### Cell culture, synchronization, drug treatment, and transfection

HeLa and HEK293T cells (from ATCC) were cultured and maintained in advanced Dulbecco’s Modified Eagle’s Medium (DMEM, Gibco) with 10% (vol/vol) fetal bovine serum (FBS, HyClone), 2 mM glutamine and 100 units/ml penicillin plus 100 μg/ml streptomycin (Invitrogen) at 37 °C with 5% CO_2_. For cell cycle synchronization, HeLa cells were synchronized at G1/S and mitosis with 2 mM thymidine and 100 ng/ml nocodazole for 16 h, respectively. CDK1 kinase inhibitor Ro3306 was added to the culture medium after 15 h of nocodazole treatment and maintained for 30 min before cell collection. Thymidine (T1895, 2 mM), nocodazole (487928, 100 ng/ml), Ro3306 (SML0569, 10 μM) were purchased from Sigma. Puromycin was purchased from Thermo (A1113802). Plasmid transfection was performed with Lipofectamine 3000 (Invitrogen) according to the manufacturer’s instructions.

### Stable cell line generation

For lentivirus production, DNA sequences of GFP-Lamin A-WT and the mutants were cloned into pLentiCMV-puro (Addgene, 39481) and were co-transfected with packing plasmids pMD2.G and psPAX2 in HEK293T using polyethyleneimine (Sigma). The supernatant containing lentivirus was collected 2 to 3 days after transfection and filtered through a 0.45 μm filter before use. The *LMNA* knockout (KO) HeLa cells were infected with GFP-Lamin A-WT and the mutant lentivirus in the presence of 8 μg/ml polybrene (Sigma) for 12 h. Twenty four hours after infection, cells were subjected to puromycin selection at 2 μg/ml for 3 days.

### Generating cell lines by CRISPR–Cas9

For the *LMNA* KO HeLa cell line, *LMNA* gene targeting single-guide RNA (sgRNA) was cloned by inserting the annealed oligos CACCggatgagatgctgcggcggg and AAACcccgccgcagcatctcatcc into pSpCas9(BB)-2A-Puro (PX459) (a gift from F. Zhang; Addgene, 48139). Twenty four hours after transfection, puromycin (2 μg/ml) was added to select for the positively transfected cells for another 72 h. The single-cell clones were isolated by fluorescence-activated cell sorting, followed by PCR and Western blotting validations.

For tagging of endogenous *LMNA* loci with monomeric fluorescently enhanced GFP, single-guide RNA (sgRNA) sequences targeting the desired genomic region (shown in Fig. S1*A*) were cloned into the PX459 vector. The homologous recombination repair template DNA was created by cloning the left (937 bp long) and right (890 bp long) homology arms, together with the GFP sequence, into the pUC19 vector. Cas9-and sgRNA-expressing plasmid (PX459) and the plasmid containing the repair template were transfected into HeLa cells using Lipofectamine 3000 (Invitrogen) according to the manufacturer’s protocol. After 24 h, the cells were selected by 2 μg/ml puromycin for several days. Single-cell clones were isolated by fluorescence-activated cell sorter (BD FACSAriaIII). Both PCR and immunoblotting were adopted to screen for the correctly edited clones.

The *LMNA* targeting sgRNA and the screening PCR primers are as follows:

sg*LMNA*: GGTGGCGCGCCGCTGGGACG.

*LMNA*fw-1 primer: AGCGCCGCACCTACACCA.

*LMNA*rv-1 primer: GCCCTGCGTTCTCCGTTTCC.

### Antibodies

The following antibodies were used in immunofluorescence analysis (IF), Western blotting analysis (WB): anti-Lamin A/C antibody (Cell Signaling Technology, 4777, 1:2000 dilution for WB, 1:200 dilution for IF); anti-Lamin A/C pSer22 antibody (Cell Signaling Technology, 13488, 1:200 for IF, 1:2000 for WB); anti-α-tubulin antibody (Sigma, T9026, 1:5000 for WB); anti-Cyclin B1 antibody (Cell Signaling Technology, 12231, 1:2000 for WB); and anti-Lamin A/C pThr19 (1:1000 for WB, 1:200 for IF). All secondary antibodies were purchased from Jackson ImmunoResearch.

The phosphorylation site-specific antibody of Lamin A/C Thr19 (T19) was produced by HuaBio company. Briefly, synthetic phosphorylated Thr19 (GAQASS-pT-PL) peptide was conjugated to rabbit albumin and immunized into rabbits as described (46). The serum was collected by a standard protocol and preabsorbed by a non-phosphorylated Lamin A peptide (GAQASSTPL) followed by affinity-purification using (GAQASS-pT-PL)-conjugated sulfone Sepharose beads. The phospho-Lamin A Thr19 site-specific antibodies were characterized according to standard procedures (47).

### Protein expression and purification

His-tagged proteins were expressed in *E. coli* strain BL21 upon induction with 1 mM isopropyl β-D-1-thiogalactopyranoside (IPTG) overnight at 16 °C. The bacteria were lysed by sonication in Ni-NTA binding buffer (50 mM NaH_2_PO_4_, pH 8.0, 500 mM NaCl, 10 mM imidazole) supplemented with a protease inhibitor cocktail (Sigma). The lysate was then incubated with Ni-NTA agarose (Qiagen) for 2 h at 4 °C. The agarose microbeads were washed three times with Ni-NTA binding buffer supplemented with 20 mM imidazole and eluted with Ni-NTA binding buffer supplemented with 200 mM imidazole. The eluted protein was dialyzed against dialysis buffer (25 mM Tris-HCl, pH 7.4, 300 mM NaCl) for 4 h at 4 °C. For purification of GST-tagged proteins, the bacteria pellets were lysed by sonication in PBS buffer containing 0.5% Triton X-100 and protease inhibitor cocktail (Sigma), followed by incubation with glutathione-agarose beads (Sigma) for 1 h at 4 °C. Then, the resin was washed three times with PBS containing 0.5% Triton X-100 before being used for pull-down assays.

### GST pull-down assays

GST-tagged recombinant proteins (10 μg) immobilized on agarose beads were incubated with 10 μg soluble recombinant proteins in PBS buffer containing 0.1% Triton X-100 at 4 °C for 4 h. The glutathione resins were then washed twice with PBS buffer containing 0.2% Triton X-100 and once with PBS buffer, followed by boiling in SDS-PAGE buffer. The samples were applied to SDS-PAGE.

### *In vitro* kinase assays

The *in vitro* kinase assay was performed following a previously reported procedure (48). Kinase reactions were carried out in 40 μl kinase buffer (20 mM Tris-HCl, pH 7.5, 10 mM MgCl_2_, 2 mM EGTA) containing 100 ng CDK1-cyclin B1 (Abcam, ab271456), 2 μg His-tagged Lamin A-WT or the mutants, 1 mM DTT, and 100 μM ATP. The mixtures were incubated at 25 °C for 10 min followed by termination by adding 5× sample buffer and boiling at 95 °C for 5 min before being resolved by SDS-PAGE and immunoblotted with the indicated antibodies.

### Immunofluorescence and time-lapse imaging

HeLa cells grown on coverslips were fixed with 3.7% formaldehyde (Thermo Fisher Scientific, 28908) in PBS for 15 min and permeabilized with PBS containing 0.1% Triton X-100 for 2 min, followed by blocking with PBST (PBS with 0.05% Tween-20) buffer containing 5% bovine serum albumin (Sigma) for 45 min at room temperature. Then the cells were incubated with primary antibodies in a humidified chamber at 4 °C overnight, followed by incubation with secondary antibodies for 1 h at room temperature. DNA was stained with DAPI (Sigma) for 2 min. Finally, the coverslips were sealed with nail polish after mounting onto a glass slide with antifade mounting medium. Images were captured by DeltaVision softWoRx software (Applied Precision) and processed by deconvolution and z-stack projection or the LSM 880 confocal microscope (Zeiss).

For time-lapse imaging, HeLa cells were cultured in glass-bottom culture dishes (MatTek) and maintained in a CO_2_-independent medium (Gibco) supplemented with 10% FBS and 2 mM glutamine. During imaging, the dishes were placed in a sealed chamber at 37 °C. Images of living cells were taken using the DeltaVision microscopy system.

### Immunoprecipitation

For GFP immunoprecipitation, *GFP-LMNA* knock-in HeLa cells were lysed in immunoprecipitation buffer (20 mM HEPES, pH 7.4, 300 mM NaCl, 1 mM EDTA, 0.1% Triton X-100) supplemented with protease inhibitor cocktail (Sigma) and phosphatase inhibitor cocktail (Sigma). The cell lysates were clarified by centrifugation and incubated with anti-GFP antibody conjugated resin (Sigma) at 4 °C with gentle rotation. After washing with lysis buffer three times for 5 min each, the GFP beads were boiled for Western blotting analysis.

### Mass spectrometry

The GFP immunoprecipitation samples and the *in vitro* phosphorylation samples were reduced with 10 mM DTT in 50 mM ammonium bicarbonate at 55 °C for 45 min, followed by alkylation with 30 mM iodoacetamide for 30 min in the dark. Subsequently, 2 μg of trypsin (Promega, V5111) was added for digestion at 37 °C overnight. After digestion, the peptides were desalted and analyzed using an Orbitrap Exploris 480 mass spectrometer. Raw files were analyzed with Proteome Discoverer 2.5. The UniProt human protein database (Proteome ID: UP000005640) was used for analysis.

### Statistics and reproducibility

All experiments were performed and repeated independently for at least three times. Statistical analyses were performed with Microsoft Excel 2013 and GraphPad Prism 8. All statistics were described in the figure legends. No statistical method was used to predetermine the sample size. Images were mounted in figures with Photoshop (Adobe) and PowerPoint.

## Data availability

All research data has been included in the manuscript.

## Supporting information

This article contains supporting information.

## Conflicts of interest

The authors declare that they have no conflicts of interest with the contents of this article.
