# Supplementary material for: Phosphorylation of Lamin A/C regulates the structural integrity of the nuclear envelope
Source: J Biol Chem. 2024 Nov 28;301(1):108033. doi: 10.1016/j.jbc.2024.108033 (PMC11731451; doi:10.1016/j.jbc.2024.108033)
Supplement: Supporting Information Figures [file mmc1.pdf]

Fig. S1

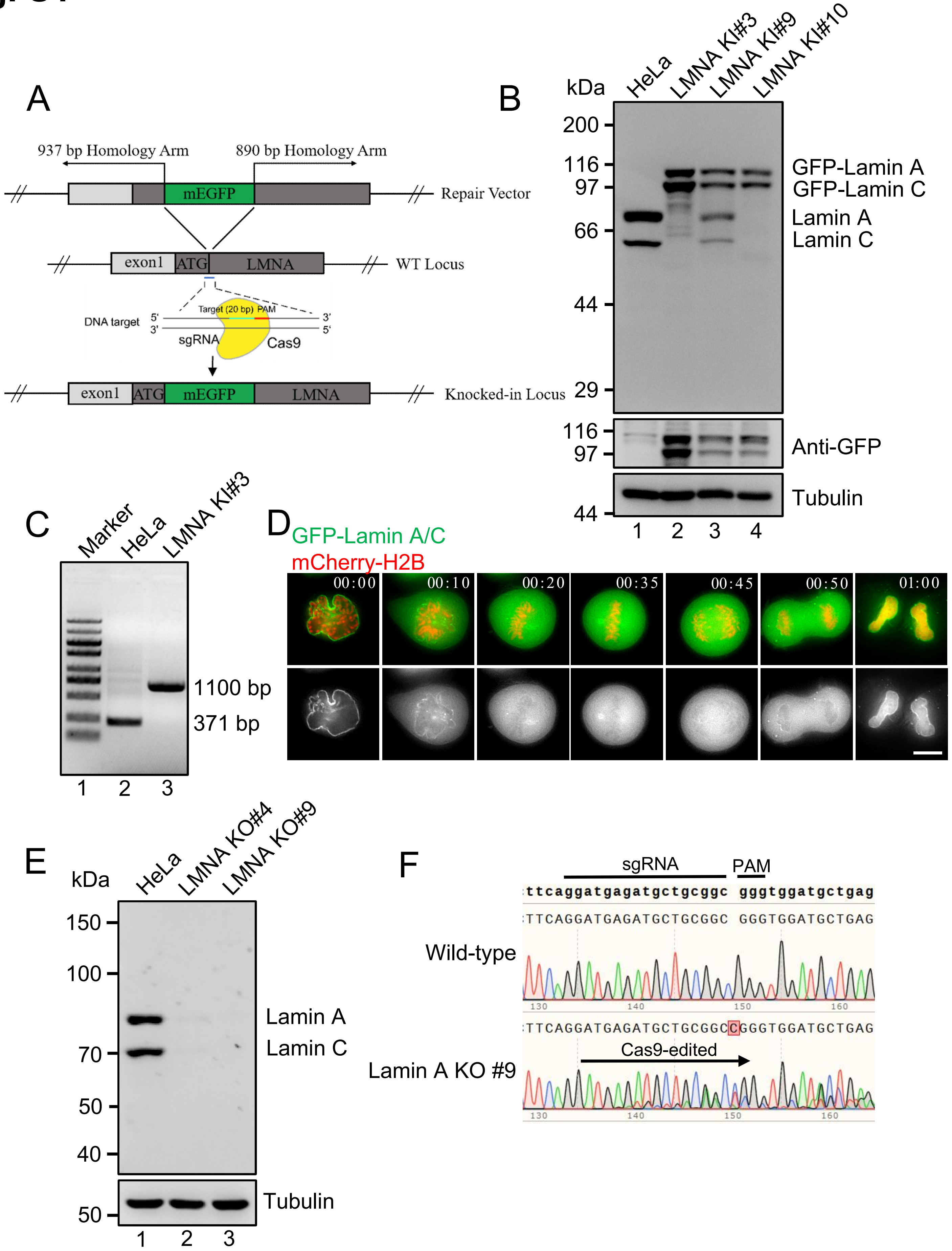
A
937 bp Homology Arm
890 bp Homology Arm
mEGFP
Repair Vector
exon1
ATG
LMNA
WT Locus
DNA target
Target (20 bp)
PAM
sgRNA
Cas9
exon1
ATG
mEGFP
LMNA
Knocked-in Locus
B
kDa
HeLa
LMNA KI#3
LMNA KI#9
LMNA KI#10
200
116
97
66
44
29
GFP-Lamin A
GFP-Lamin C
Lamin A
Lamin C
116
97
Anti-GFP
Tubulin
44
1 2 3 4
C
Marker
HeLa
LMNA KI#3
1100 bp
371 bp
1 2 3
D
GFP-Lamin A/C
mCherry-H2B
00:00
00:10
00:20
00:35
00:45
00:50
01:00
E
kDa
HeLa
LMNA KO#4
LMNA KO#9
150
100
70
50
40
Lamin A
Lamin C
Tubulin
50
1 2 3
F
sgRNA
PAM
ttcaggatgagatgctgcggc gggtggatgctgag
TTCAGGATGAGATGCTGCGGC GGGTGGATGCTGAG
Wild-type
130 140 150 160
TTCAGGATGAGATGCTGCGGCCGGGTGGATGCTGAG
Cas9-edited
Lamin A KO #9
130 140 150 160

Figure S1 Generation of mEGFP-Lamin A/C knock-in and knock out HeLa cell lines by CRISPR-Cas9.

(A) Schematic representation of the engineering design of the endogenous *LMNA* gene with an mEGFP tag via CRISPR-Cas9 mediated gene editing to generate mEGFP-Lamin A/C knock-in HeLa cell line. The mEGFP was introduced into the sequence following the TAG codon of *LMNA* gene.

(B) The mEGFP-Lamin A/C knock-in HeLa cells were validated through Western blotting using an anti-Lamin A/C and anti-GFP antibody.

(C) Genotype of the mEGFP-Lamin A/C knock-in single HeLa cell clone by PCR.

(D) Real-time imaging of mEGFP-Lamin A/C knock-in HeLa cells. The chromosome was marked by H2B-mCherry. Scale bar, 10 μm.

(E) The *LMNA* knock out HeLa cells were validated through Western blotting using an anti-Lamin A/C antibody.

(F) Genotype of *LMNA* knock out single HeLa cell clone by sanger sequencing.

Fig. S2

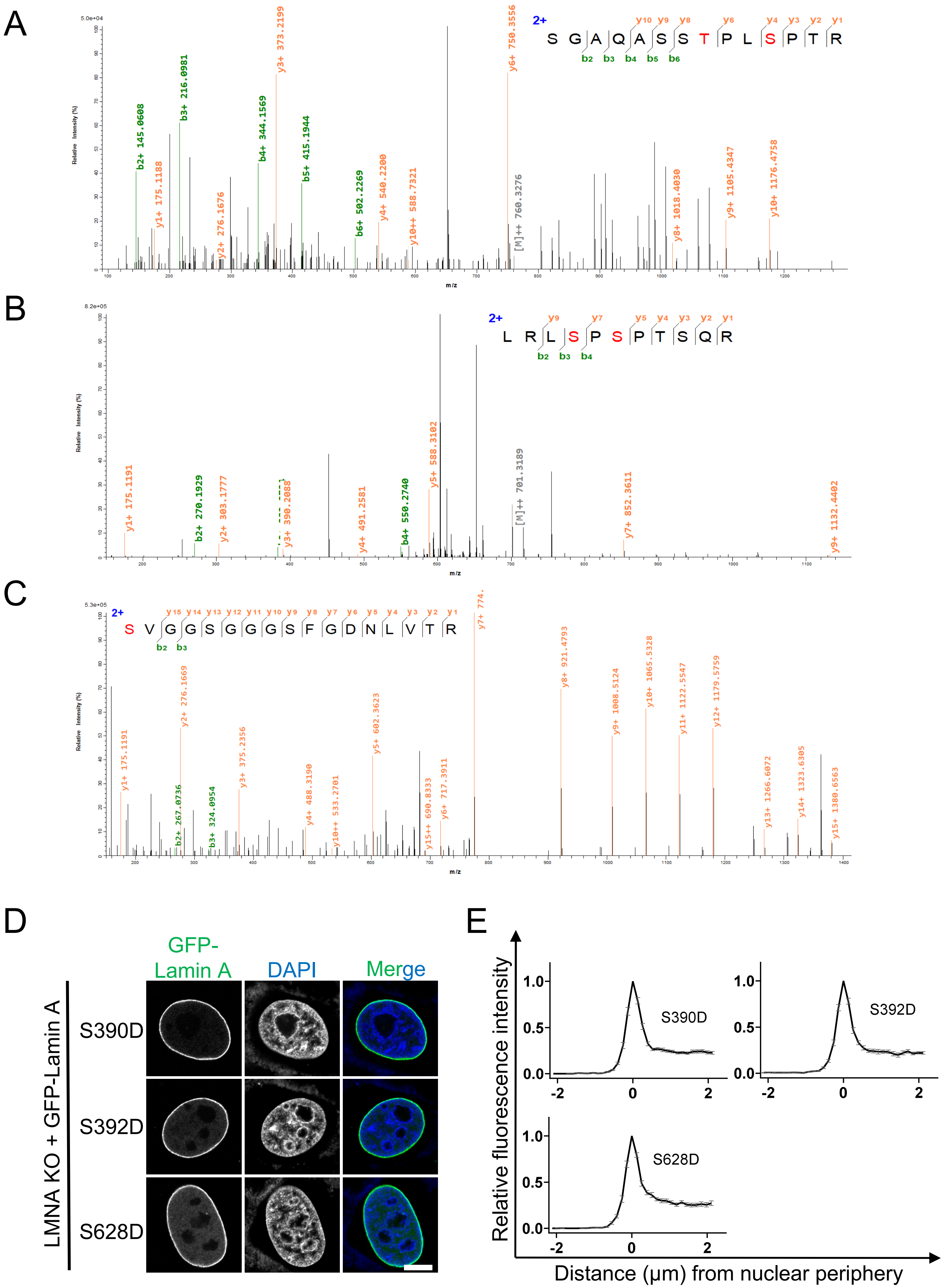

A
2+
S G A Q A S S T P L S P T R
y10 y9 y8 y6 y4 y3 y2 y1
b2 b3 b4 b5 b6
5.0e+04
Relative Intensity (%)
b2+ 145.0608
y1+ 175.1188
b3+ 216.0981
y2+ 276.1676
b4+ 344.1569
y3+ 373.2199
b5+ 415.1944
b6+ 502.2269
y4+ 540.2200
y10++ 588.7321
y6+ 750.3556
[M]++ 760.3276
y8+ 1018.4030
y9+ 1105.4347
y10+ 1176.4758
m/z
B
2+
L R L S P S P T S Q R
y9 y7 y5 y4 y3 y2 y1
b2 b3 b4
8.2e+05
Relative Intensity (%)
y1+ 175.1191
b2+ 270.1929
y2+ 303.1777
y3+ 390.2088
y4+ 491.2581
b4+ 550.2740
y5+ 588.3102
[M]++ 701.3189
y7+ 852.3611
y9+ 1132.4402
m/z
C
2+
S V G G S G G G S F G D N L V T R
y15 y14 y13 y12 y11 y10 y9 y8 y7 y6 y5 y4 y3 y2 y1
b2 b3
5.3e+05
Relative Intensity (%)
y1+ 175.1191
b2+ 267.0736
y2+ 276.1669
b3+ 324.0954
y3+ 375.2356
y4+ 488.3190
y10++ 533.2701
y5+ 602.3623
y15++ 690.8333
y6+ 717.3911
y7+ 774.
y8+ 921.4793
y9+ 1008.5124
y10+ 1065.5328
y11+ 1122.5547
y12+ 1179.5759
y13+ 1266.6072
y14+ 1323.6305
y15+ 1380.6563
m/z
D
GFP-Lamin A
DAPI
Merge
LMNA KO + GFP-Lamin A
S390D
S392D
S628D
E
Relative fluorescence intensity
Distance (μm) from nuclear periphery
S390D
S392D
S628D

Figure S2 Phosphorylation of Lamin A perturbs its correct subcellular localization.

(A) Mass spectrum of Lamin A Thr19 and Ser22 phosphorylation in mitosis.

(B) Mass spectrum of Lamin A Ser390 and Ser392 phosphorylation in mitosis.

(C) Mass spectrum of Lamin A Ser628 phosphorylation in mitosis.

(D) Representative HeLa cells expressing GFP-tagged Lamin A mutants, in which endogenous LMNA was knocked out (KO), were fluorescently imaged and shown. Scale bar, 10 μm.

(E) Line scan analysis was performed for GFP-Lamin A mutants. The immunofluorescence intensity along the 4-μm lines that were drawn across the nuclear periphery (0 μm) was statistically analyzed. The negative x coordinates indicate positions outside the nucleus. n=20.

Fig. S3

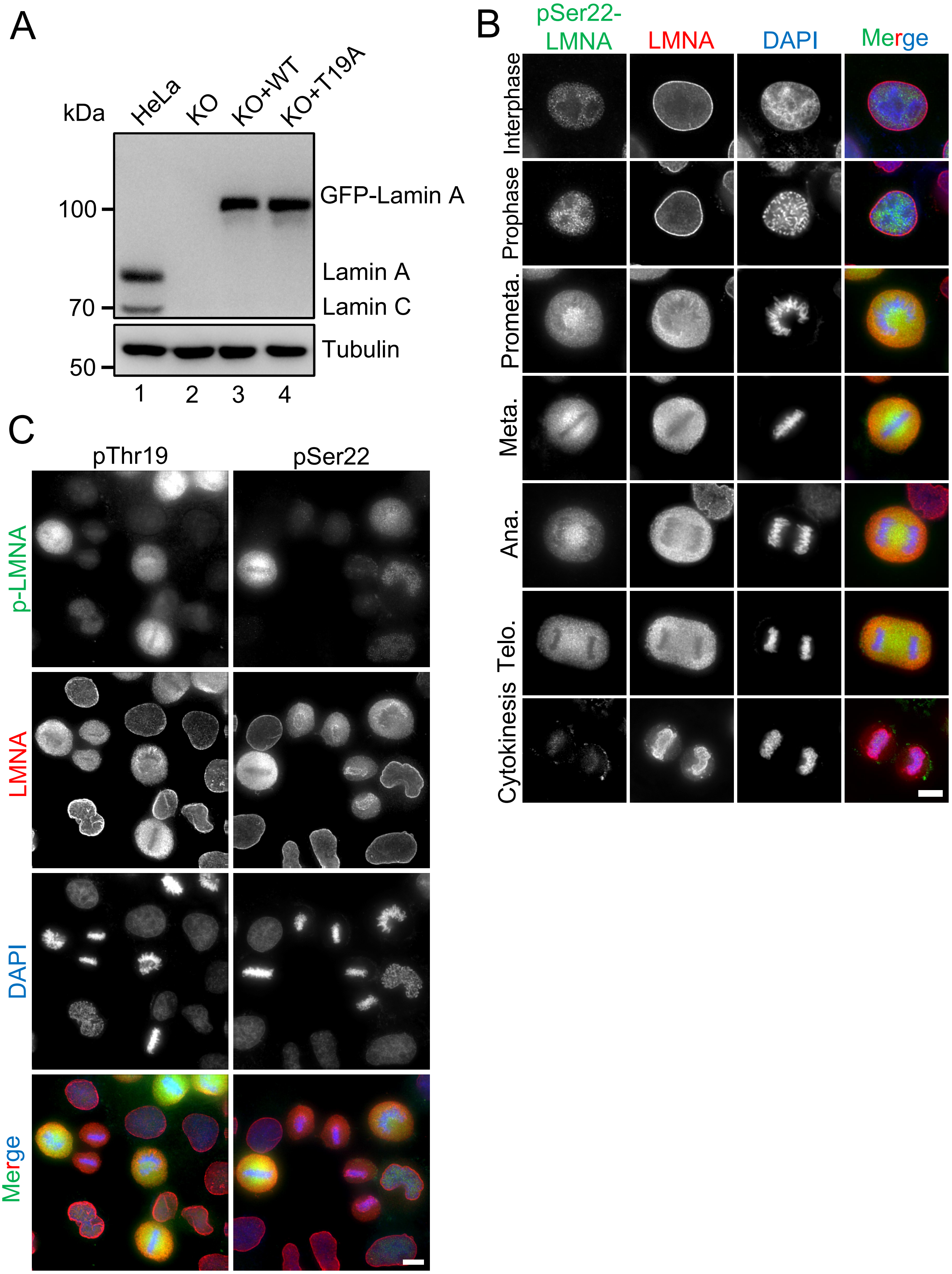
A
kDa
HeLa
KO
KO+WT
KO+T19A
100
70
50
GFP-Lamin A
Lamin A
Lamin C
Tubulin
1
2
3
4
B
pSer22-LMNA
LMNA
DAPI
Merge
Interphase
Prophase
Prometa.
Meta.
Ana.
Telo.
Cytokinesis
C
pThr19
pSer22
p-LMNA
LMNA
DAPI
Merge

Figure S3 Thr19 of Lamin A/C is phosphorylated in mitosis.

(A) HeLa cells stably expressing GFP-Lamin A-WT and the T19A mutant, in which endogenous LMNA was inactivated. Western blotting analysis was employed to assess the level of Lamin A protein.

(B) HeLa cells were immunofluorescently stained for pSer22-Lamin A/C (green) and Lamin A/C (red), and nucleus (DAPI, blue). Representative images of the cells at the various cell cycle stages were shown.

(C) Wide-field immunofluorescence staining for pThr19-Lamin A/C and pSer22-lamin A/C.

Fig. S4

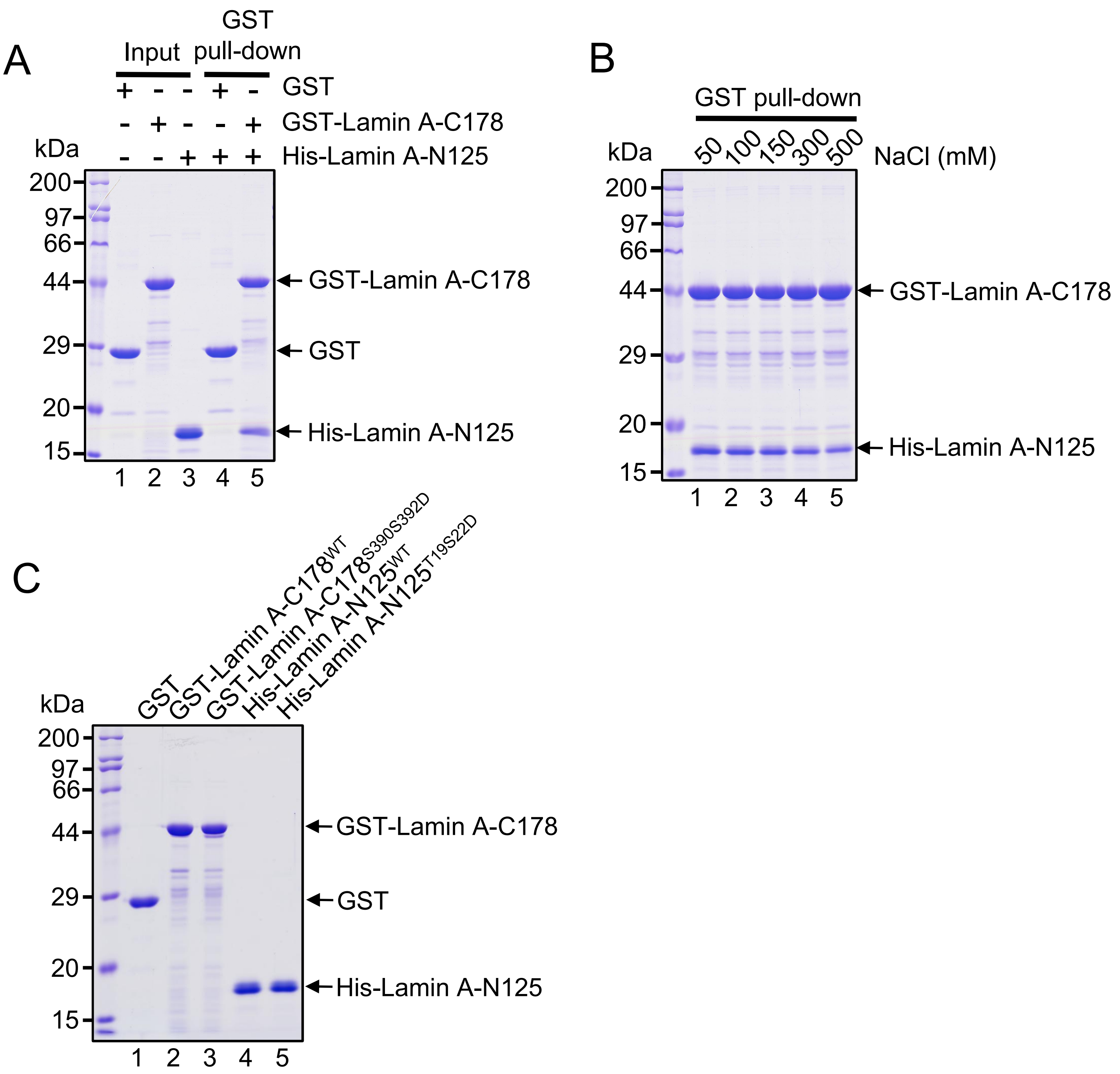
A
Input
GST pull-down
GST
GST-Lamin A-C178
His-Lamin A-N125
kDa
200
97
66
44
29
20
15
GST-Lamin A-C178
GST
His-Lamin A-N125
1 2 3 4 5
B
GST pull-down
50 100 150 300 500 NaCl (mM)
kDa
200
97
66
44
29
20
15
GST-Lamin A-C178
His-Lamin A-N125
1 2 3 4 5
C
GST
GST-Lamin A-C178WT
GST-Lamin A-C178S390S392D
His-Lamin A-N125WT
His-Lamin A-N125T19S22D
kDa
200
97
66
44
29
20
15
GST-Lamin A-C178
GST
His-Lamin A-N125
1 2 3 4 5

Figure S4 Biochemical analysis of the Lamin A-C178 and Lamin A-N125 interaction using GST pull-down assay.

(A) Recombinant GST and GST-tagged Lamin-A-C178-WT were attached to the glutathione microbeads, were used as matrices to absorb Lamin A-N125-WT. Following three washes, the eluates from the microbeads were applied to SDS-PAGE electrophoresis and Coomassie Brilliant Blue staining.

(B) Dissociation of the Lamin A-C178 and Lamin A-N125 interaction by increasing salt concentration.

(C) Characterization of recombinant purified proteins used in Figure 6B. Recombinant proteins were subjected to SDS-PAGE electrophoresis and Coomassie Brilliant Blue staining.
